# Genipin Inhibits TNF-α-Induced Vascular Smooth Muscle Cell Proliferation and Migration via Induction of HO-1

**DOI:** 10.1371/journal.pone.0074826

**Published:** 2013-08-27

**Authors:** Fengrong Jiang, Rilei Jiang, Xiaojia Zhu, Xu Zhang, Zhan Zhan

**Affiliations:** Discipline of Chinese and Western Integrative Medicine, Nanjing University of Chinese Medicine, Nanjing, China; University of Kansas Medical Center, United States of America

## Abstract

Vascular smooth muscle cell (VSMC) proliferation and migration triggered by inflammatory stimuli contributes importantly to the pathogenesis of atherosclerosis and restenosis. On the other hand, genipin, an aglycon of geniposide, exhibits diverse pharmacological functions such as antitumor and anti-inflammatory effects. The protective effects of genipin on the cardiovascular system have also been reported. However, the molecular mechanism involved remains unknown. This study aimed to elucidate the precise function of genipin in VSMCs, focusing particularly on the role of heme oxygenase-1 (HO-1), a potent anti-inflammatory enzyme. We found that pretreatment of genipin induced HO-1 mRNA and protein levels, as well as its activity in VSMCs. Genipin inhibited TNF-α-induced VSMC proliferation and migration in a dose-dependent manner. At the molecular level, genipin prevented ERK/MAPK and Akt phosphorylation while left p38 MAPK and JNK unchanged. Genipin also blocked the increase of ROS generation induced by TNF-α. More importantly, the specific HO-1 siRNA partially abolished the beneficial effects of genipin on VSMCs. These results suggest that genipin may serve as a novel drug in the treatment of these pathologies by inducing HO-1 expression/activity and subsequently decreasing VSMC proliferation and migration.

## Introduction

The abnormal proliferation and migration of vascular smooth muscle cells (VSMCs) are critical events in the pathogenesis of atherosclerosis and restenosis [1]. One of the risk factors for these cardiovascular diseases is chronic and mild inflammation occurred in arteries [2]. Quiescent VSMCs stay in a “contractile” state when unchallenged, however, in response to various stimuli including inflammatory cytokine stimulation, VSMCs will switch to an active “synthetic” state, leading to uncontrolled proliferation and migration [3,4]. TNF-α is one of the powerful inflammatory cytokines and robustly promotes the secretion of multiple chemokines and upregulates adhesion molecule expression, as well as recruiting monocytes to injury site and enhancing their adhesiveness to the endothelium [5]. TNF-α also regulates VSMC proliferation, migration and synthetic behavior [6] and induces the differentiation of VSMCs into osteoblast/chondrogenic cells, which causing calcium deposition and increasing arterial stiffness [7]. Therefore, inhibition of arterial inflammation and the subsequent VSMC proliferation and migration has high value in the treatment of atheroslcerosis and restenosis [8,9].

**Figure 1:**
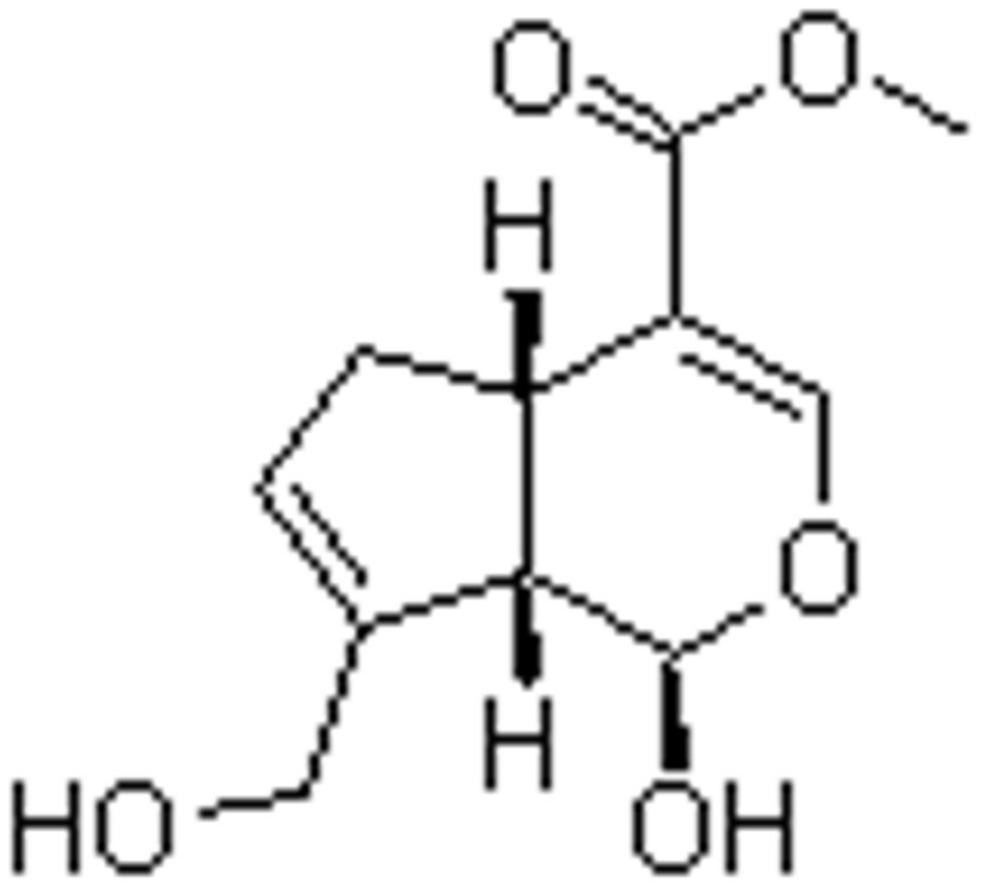
Chemical structure of genipin.

Genipin (Figure 1), the metabolite of geniposide, is a natural product present in fruit of *Gardenia* *jasminoides* [10]. Previous study shows that geniposide is absorbed and transformed to genipin in the bowel, indicating that genipin may be the major form of geniposide in blood [11]. Genipin is pharmacologically active against β-cell dysfunction through inhibiting UCP2-mediated proton leak [12]. It also has anti-inflammatory, anti-oxidative and neuroprotective activities [13–15]. Particularly, some studies also present the beneficial effects of genipin on cardiovascular system. For example, genipin attenuated thrombotic occlusion induced by photochemical reaction in the mouse femoral artery and inhibited collagen-induced platelet aggregation [16]. However, there’s no study reporting the pharmaceutical action of genipin and its underlying mechanism in VSMCs.

Endogenous antioxidant defense is activated to protect cells against injury associated with oxidative stress. Heme oxygenase-1 (HO-1) plays an essential role in this process. HO-1 is a cytoprotective enzyme that degrades heme (a potent oxidant) to generate carbon monoxide (CO, a vasodilatory gas that has anti-inflammatory properties), bilirubin (an antioxidant derived from biliverdin), and iron (sequestered by ferritin) [17]. The beneficial effects of HO-1 upregulation in anti-atherosclerosis, anti-diabetes and renoprotection have been reported in a series of animal models [18]. In addition, HO-1 protects VSMCs from oxidative injury and antagonizes VSMC proliferation and migration [19,20]. Importantly, the ability of genipin to increase HO-1 expression and activity has been revealed in RAW264.7 macrophages [21]. Based on these observations mentioned above, we carried out the present study to examine whether genipin inhibits VSMC proliferation and migration during inflammation through induction of HO-1.

## Results

### Genipin increases HO-1 expression and activity in VSMCs

**Figure 2:**
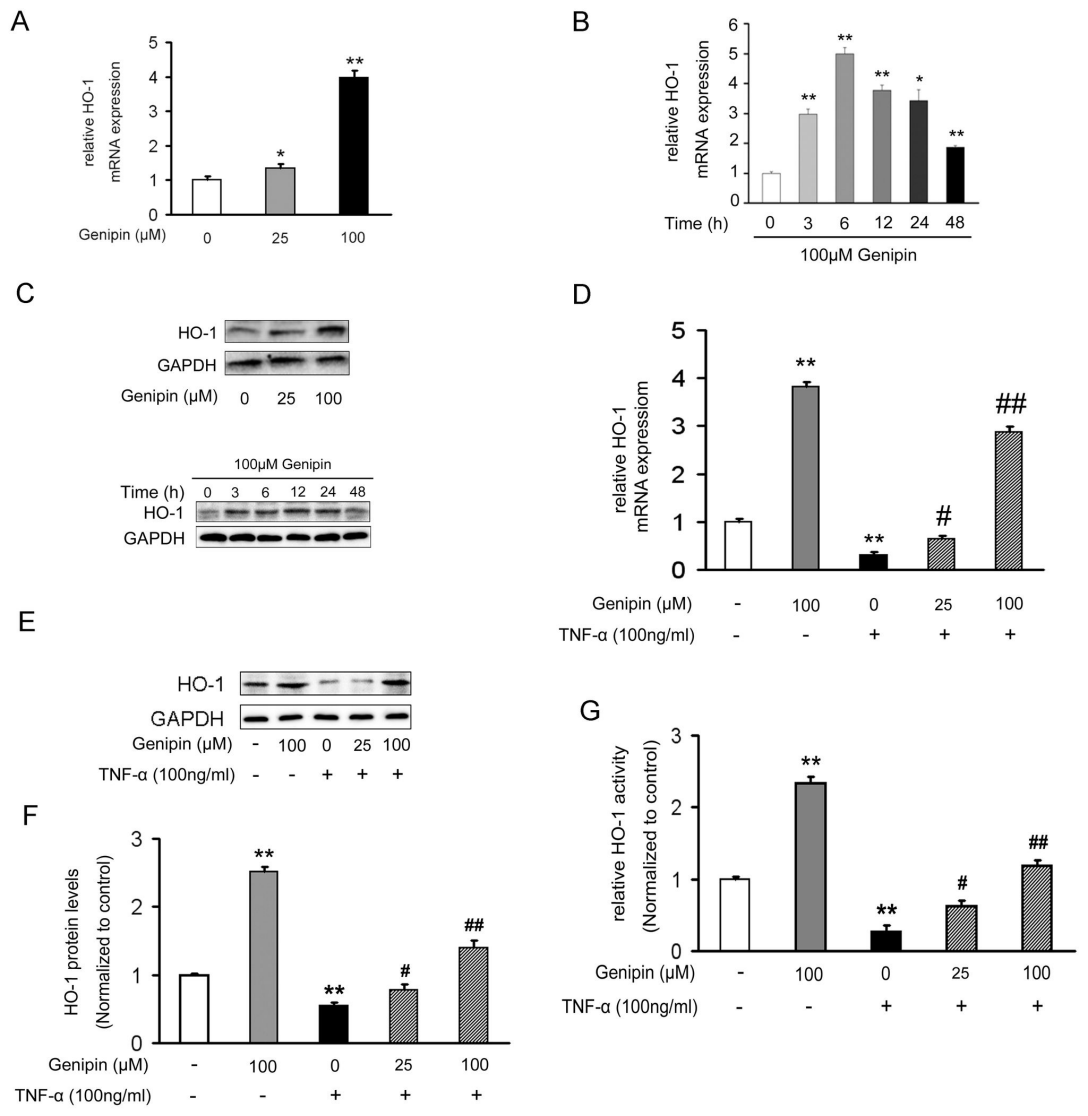
Regulation of HO-1 expression and activity by genipin in VSMCs. Cells were treated with indicated concentrations of genipin for 24 h (A) or with 100 μM genipin for indicated time-points (B). For the combination of genipin and TNF-α, cells were pretreated with 100 μM genipin for 1 h and then stimulated with 100 ng/ml TNF-α for 24 h (D–F). HO-1 mRNA and protein expression levels were respectively determined by RT-qPCR (A–B and D) and western blot (C and E–F). HO-1 activity was assessed by a biochemical analysis (G). Results are average of three separate experiments. Data are reported as means ± SD. *P<0.05; **P<0.01 compared with the control group; ^*#*^ *P*<0.05; ^*# #*^ *P*<0.01 compared with the TNF-α-treated group.

To detect the effects of genipin on basal HO-1 expression in VSMCs, 25 or 100 μM genipin was added to VSMCs, respectively. As shown in Figure 2A, genipin dose-dependently increased HO-1 expression in VSMCs. The induction of HO-1 mRNA expression peaked at 6 h (5 folds) and then declined gradually (Figure 2B). Consistent with mRNA expression, HO-1 protein expression showed similar trends (Figure 2C). In contrast, stimulation of VSMCs with 100 ng/ml TNF-α for 24 h inhibited HO-1 mRNA (Figure 2D) and protein (Figure 2E and 2F) expression levels, as well as its enzymatic activity (Figure 2G). Moreover, pretreating cells with genipin markedly blocked the inhibitory effects of TNF-α on HO-1 expression and activity (Figure 2D–G).

### Genipin inhibits TNF-α-induced VSMC proliferation and retards cell cycle progression

**Figure 3:**
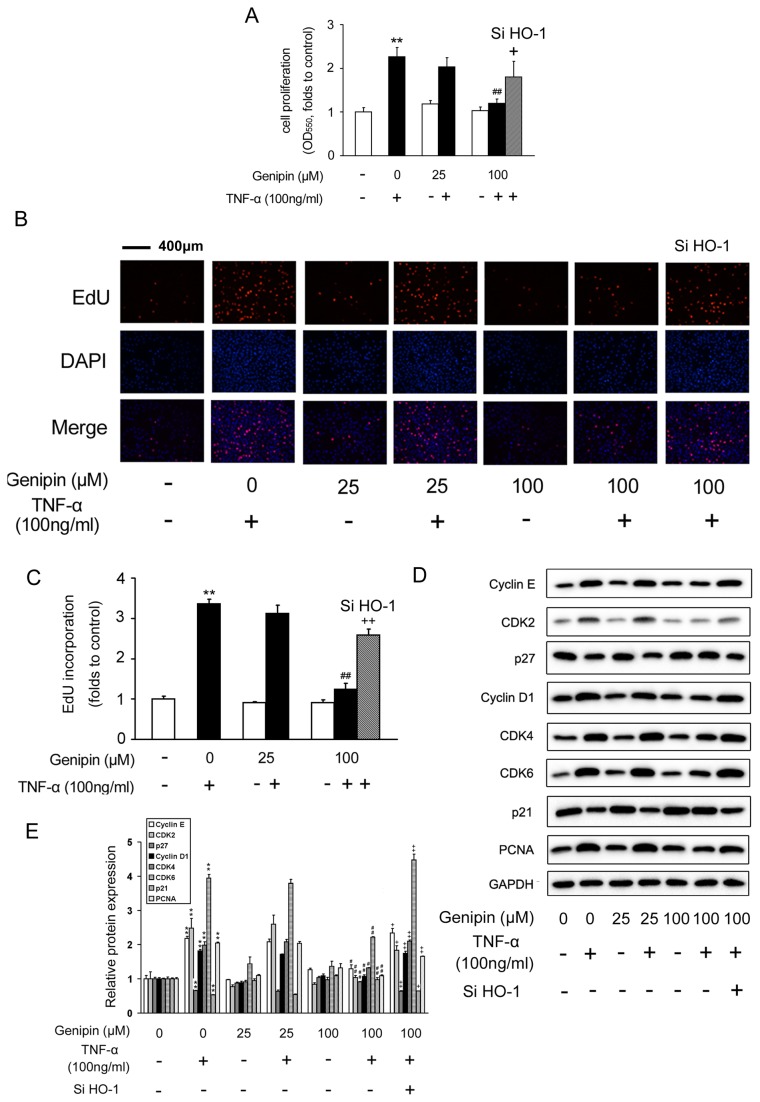
Inhibition of TNF-α-induced VSMC proliferation by genipin. Cells were pretreated with 100 μM genipin for 1 h and then stimulated with 100 ng/ml TNF-α for 24 h. HO-1 specific siRNA was added to cells for 24 h in advance when required. VSMC proliferation was determined by MTT assay (A) and EdU (B and C). (D) Western blot analysis of the protein levels of key regulators involved in the cell cycle progression. GAPDH was used as an internal control. A representative image was shown from three separate experiments. (E) Quantitative data of Figure 3D. Data are presented as means ± SD. *P<0.05, **P<0.01 compared with the control group; ^*#*^ *P*<0.05, ^*# #*^ *P*<0.01 compared with the TNF-α-treated group; ^*+*^ *P*<0.05; ^*+ +*^ *P*<0.01 compared with the group treated with genipin and TNF-α.

To dissect the effects of genipin on VSMC growth and proliferation, MTT assay was performed and we found that genipin alone did not affect the basal level of VSMC growth (Figure 3A). However, genipin (25 or 100 μM) blocked TNF-α induced VSMC proliferation. 100 μM of genipin showed the strongest inhibitory effect (*P*<0.01 *vs.* TNF-α) (Figure 3A). To explore whether these effects of genipin were dependent on HO-1 induction, we knocked down HO-1 expression with HO-1 specific siRNA oligos (the knockdown efficiency was shown in Figure S1) and found that the inhibitory effect of genipin on VSMC proliferation was attenuated (Figure 3A). The results from MTT assay were confirmed by EdU incorporation assay (Figure 3B-C, the pink dots represent EdU-incorporated nuclei).

Cell cycle progression is tightly associated with accelerated cellular proliferation. As shown in Figure 3D, genipin dramatically reduced PCNA protein expression, verifying its inhibitory effects on VSMC proliferation. On the other hand, cell cycle progression is controlled by cyclins and cyclin-dependent kinases (CDKs). We found that TNF-α-induced protein expression levels of cyclin D1, cyclin E, as well as CDK2/4/6, were all suppressed by genipin in a concentration-dependent manner.

In contrast, p21 and p27 are negative regulators of the cyclin-CDK complexes, and their protein expression was correspondingly induced by genipin. However, all these changes were partially abolished by HO-1 siRNA oligos (Figure 3D and 3E).

### Genipin inhibits TNF-α-induced VSMC migration

**Figure 4:**
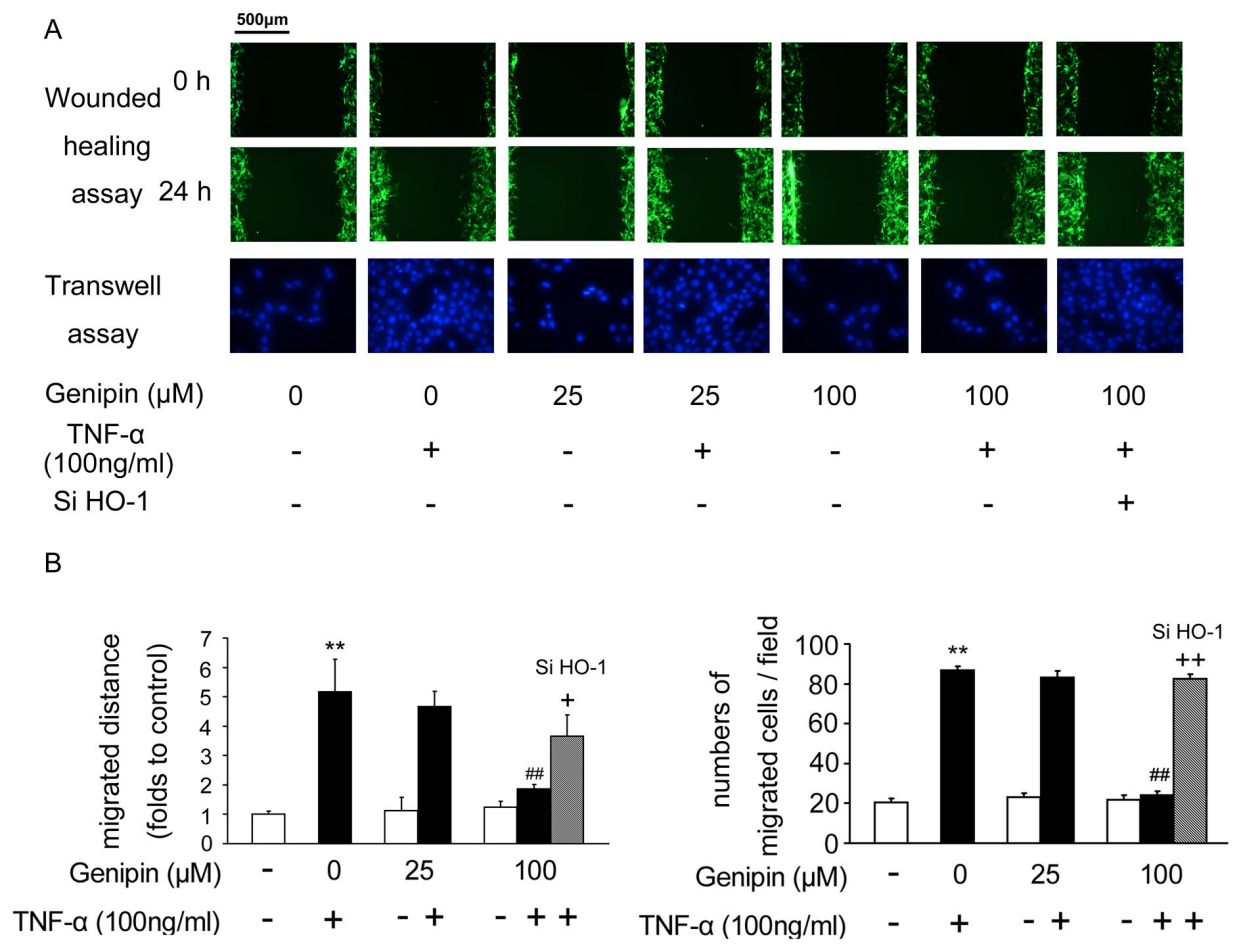
Inhibition of TNF-α-induced VSMC migration by genipin. Cells were treated as described in Figure 3. (A) VSMC migration was assessed by the wounded healing assay and transwell assay. (B) Quantitative analysis of (A). Data represent the means ± SD from three independently prepared samples. **P<0.01 compared with the control group; ^*# #*^ *P*<0.01 compared with the TNF-α-treated group; ^*+*^ *P*<0.05; ^*+ +*^ *P*<0.01 compared with the group treated with genipin and TNF-α.

To evaluate the effects of genipin on quiescent and TNF-α-induced VSMC migration, we performed wounded healing and transwell assay. To help witness the migrated cells clearly, we infected VSMCs with adenoviruses expressing GFP protein. This manipulation did not alter cell viability (Figure S2). In Figure 4, genipin (25 or 100 μM) showed no effect on quiescent VSMC migration. However, TNF-α-induced VSMC migration was inhibited by genipin (*P*<0.01 *vs.* TNF-α). Similarly, knockdown of HO-1 partially blocked the inhibitory effects of genipin on VSMC migration.

**Figure 5:**
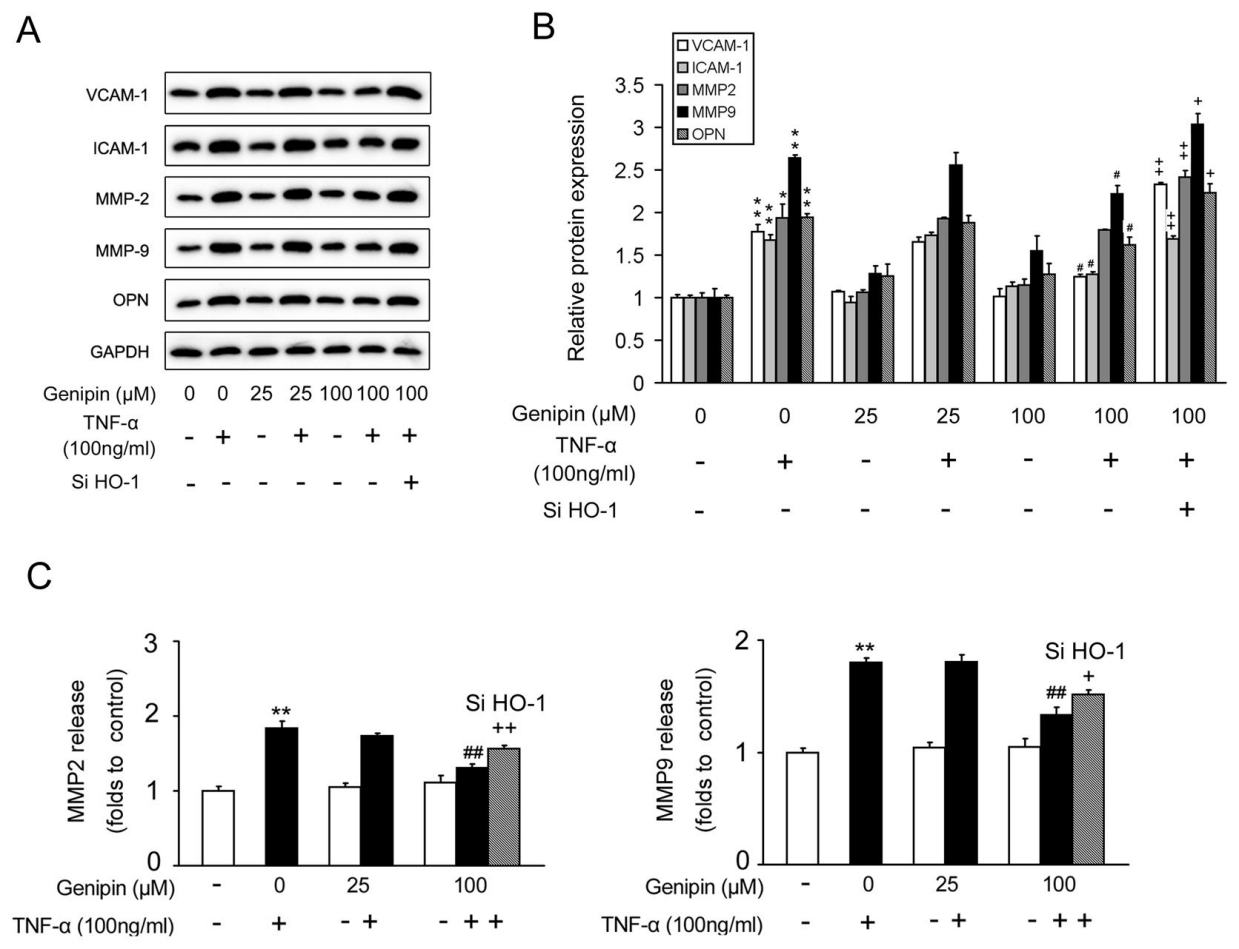
Inhibition of migration-associated regulators in VSMCs by genipin. Cells were treated as described in Figure 3. (A) Western blot analysis for the protein levels involved in the cell migration. (B) Quantitative data of Figure 5A. (C) ELISA analysis for the concentrations of MMP-2 and MMP-9 in the culture medium. Data are presented as means ± SD from three independent experiments. **P<0.01 compared with the control group; ^*# #*^ *P*<0.01 compared with the TNF-α-treated group; ^*+*^ *P*<0.05; ^*+ +*^ *P*<0.01 compared with the group treated with genipin and TNF-α.

Intercellular adhesion molecule 1 (ICAM-1), vascular cell adhesion molecule 1 (VCAM-1), matrix metalloproteases (MMPs) and osteopontin (OPN) are key regulators involved in cell adhension and inflammation. As shown in Figure 5A and 5B, genipin pretreatment inhibited the upregulation of all these proteins induced by TNF-α in a dose-dependent manner. In addition, ELISA assay showed that genipin decreased the concentration of MMP-2 and MMP-9 released to the medium, consistent with the decrease in MMP-2 and MMP-9 protein levels (Figure 5C).

### Genipin inhibits TNF-α-induced kinase activation

**Figure 6:**
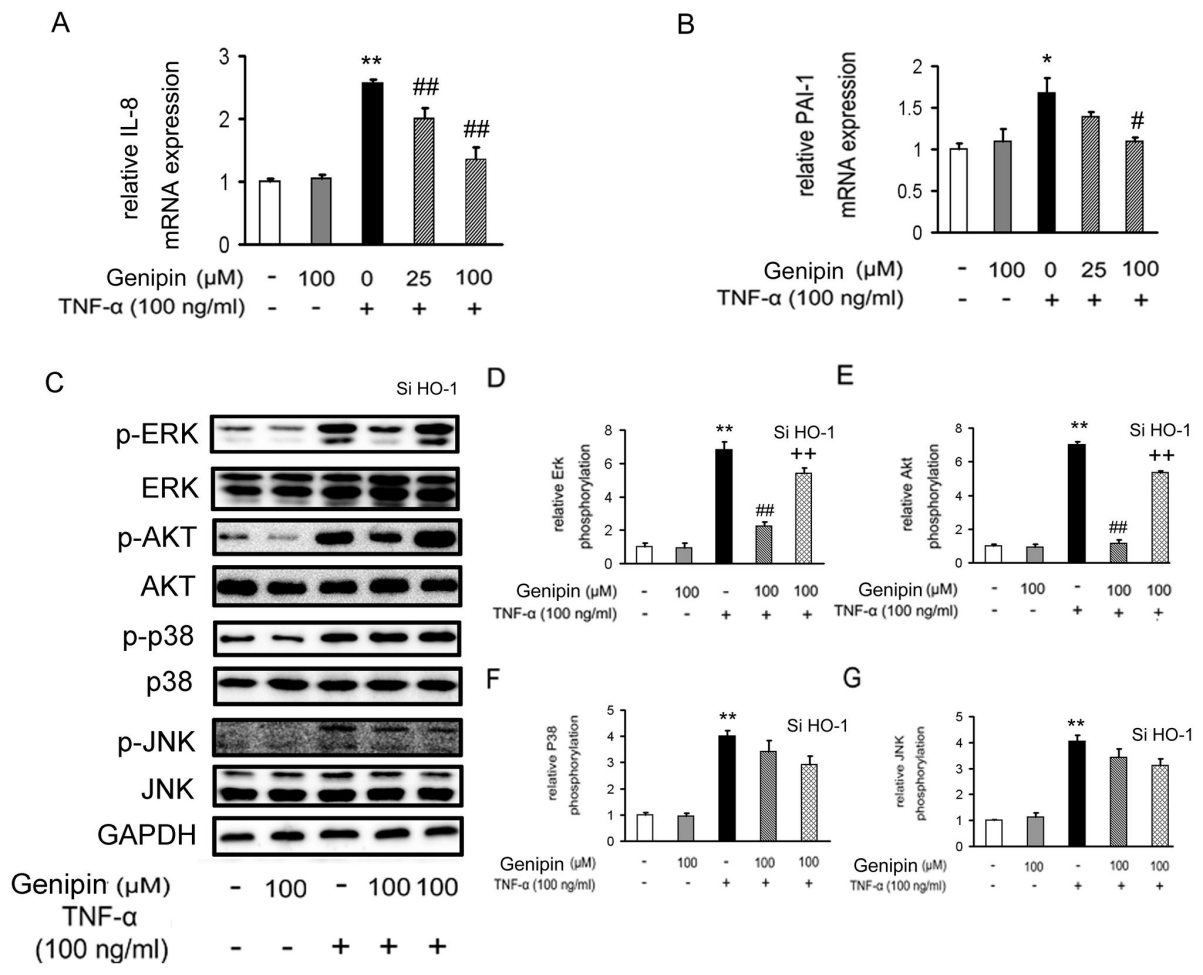
Inhibition of TNF-α-induced kinase activation by genipin. Cells were treated as described in Figure 3 and mRNA expression levels of IL-8 and PAI-1 were assessed by RT-qPCR (A and B). ERK1/2, AKT, p38 and JNK phosphorylation was determined by Western blot analysis (C–G). Note the stimulation time of TNF-α was shortened to 15 min in these experiments. Data are presented as means ± SD from five independent experiments. *P<0.05; **P<0.01 compared with the control group; ^*#*^ *P*<0.05; ^*# #*^ *P*<0.01 compared with the TNF-α-treated group; ^*+*^ *P*<0.05; ^*+ +*^ *P*<0.01 compared with the group treated with genipin and TNF-α.

IL-8 and PAI-1 are two important factors involved in the vascular inflammation. In our study, we found that the mRNA expression levels of the two inflammatory factors were significantly higher in TNF-α-treated VSMCs. On the contrary, genipin inhibited their expression in a dose-dependent manner (Figure 6A and 6B). In addition, studies demonstrated that the mitogen-activated protein kinases including ERK1/2, serine/threonine kinase Akt and p38, as well as JNK, are intensively involved in the inflammation and VSMC proliferation and migration. As expected, phosphorylated (the active form) ERK1/2, Akt, p38 and JNK were all increased in TNF-α-treated VSMCs (Figure 6C–G). In contrast, administration of genipin markedly blocked ERK1/2 and Akt phosphorylation, while left p38 and JNK phosphorylation unchanged. Once again, the inhibitory effects of genipin on Erk and Akt activations were attenuated when HO-1 was knocked down. Lastly, we evaluated the roles of MAPK and PI3K/Akt signaling pathways in genipin-induced HO-1 expression by using specific inhibitors. As shown in Figure S3, PD98059 (an ERK1/2 inhibitor) and Wortmannin (an Akt inhibitor) partially abrogated the increase of HO-1 protein expression induced by genipin, but SB203580 (a p38 MAPK inhibitor) and SP600125 (a JNK inhibitor) failed to do that, indicating that ERK and Akt are dominant signaling pathways mediating genipin-induced HO-1 expression.

### Genipin inhibits TNF-α-induced ROS generation in VSMCs

**Figure 7:**
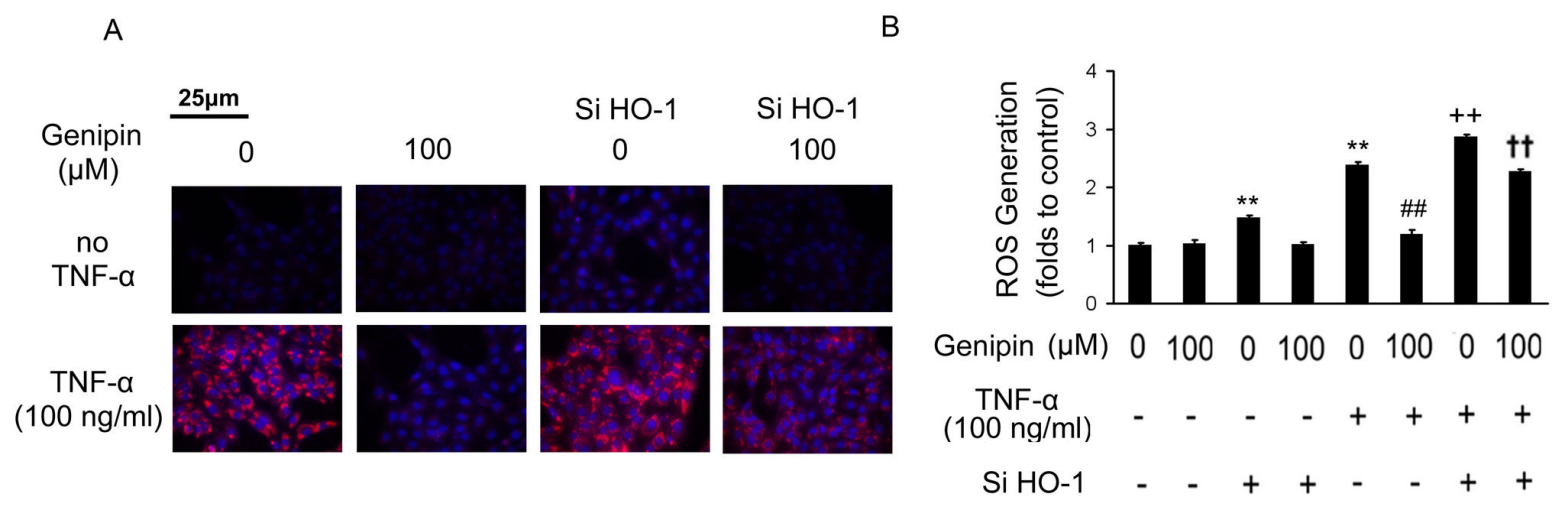
Suppression of ROS generation in VSMCs by genipin. VSMCs were pretreated with 100 μM genipin for 24 h and then stimulated with 100 ng/ml TNF-α for 30 minutes. (A) Detection of ROS generation by MitoTracker Red probe staining. (B) Quantitative data of three independent experiments are expressed as fold increase compared with control. Data are presented as means ± SD. **P<0.01 compared with the control group; *^# #^ P*<0.01 and *^+ +^ P* < 0.01compared with the TNF-α-treated group; ^† †^ *P*<0.01 compared with the group treated with HO-1 siRNA and TNF-α.

To directly evaluate the effect of genipin on ROS production, we evaluated ROS levels in TNF-α-treated VSMCs. As shown in Figure 7A and 7B, genipin alone did not change the basal level of ROS generation, while treatment with TNF-α (100 ng/ml) for 30 min caused a greater increase of red fluorescence compared with control cells. However, pretreatment with genipin (100 μM) for 1 h significantly inhibited ROS generation. As expected, knockdown of HO-1 attenuated such inhibitory effects.

## Discussion

In the present study, we showed that genipin inhibits TNF-α-induced VSMC proliferation and migration, activation of Erk/MAPK and Akt pathways as well as ROS production. Furthermore, we identified HO-1 as one of the major mediators in the protective functions of genipin.

During the progression of atherosclerosis, chronic and mild inflammation and oxidative stress play a crucial role in triggering VSMC dysfunction. To eliminate the oxidative stress, endogenous antioxidant defense including HO-1 induction is evoked. The beneficial effects of HO-1 upregulation in anti-diabetes, anti-atherosclerosis and renoprotection have been reported in a series of animal models [18]. Anti-atherosclerotic effect of HO-1 induction is mediated by various mechanisms, including the blockade of innate and adaptive immune response and increased production of CO. HO-1 protects VSMCs from oxidative injury and antagonizes VSMC proliferation [19,20]. In the present study, HO-1 expression was decreased after 24 h treatment of TNF-α, as well as its activity, indicating a heavier oxidative burden in VSMCs. On the contrary, genipin induced HO-1 expression and its activity in VSMCs. Consistent with our results, genipin also increases HO-1 expression and shows anti-inflammatory capacity in RAW264.7 macrophages [21]. All these findings suggest that genipin is an inducer of HO-1 and such induction may be independent of cell types.

Cell cycle is a major convergent point in VSMC proliferation. Such process is controlled by multiple protein kinases, including catalytic CDKs and regulatory Cyclins [22]. In addition, p21 and p27 are negative regulators of the protein kinases and cyclins, therefore blocking the cell cycle at G_0_/G_1_ phase [23]. In the present study, genipin inhibited CDK2/4/6 and Cyclin D1/E, as well as PCNA expression. In contrast, the protein levels of p21 and p27 were correspondingly increased. Based on these findings, it is reasonable to speculate that the inhibitory effects of genipin on VSMC proliferation are mediated by the induction of a cell cycle arrest.

Chronic inflammation is intensively involved in cardiovascular disease. In response to inflammatory stimuli, Erk1/2 phosphorylation is increased and subsequently promotes VSMC proliferation and migration [24]. Such process is mediated by the activation of PI3K and its downstream target Akt [25], which is an important regulator involved in cell metabolism, growth and vascular remodeling [26]. Our study showed that OPN, a chemotactic protein closely associated with VSMC inflammation, calcification and migration, was decreased by pretreatment with genipin. Other inflammatory cytokines, such as IL-8 and PAI-1, were also reduced by genipin. in addition, pretreatment of genipin inhibited TNF-α-induced Erk1/2 and Akt phosphorylation. More importantly, all these effects of genipin were abolished by HO-1 knockdown. Our findings clearly demonstrate that HO-1 induction at least dominantly, if not totally, mediates genipin-induced deactivation of VSMCs. Taken together, genipin has profound effects on various pathways involved in VSMC pathological activation and HO-1 is one of the major targets of genipin.

It should be noted that genipin is an inhibitor for uncoupling protein-2 (UCP2) [12]. UCP2 is ubiquitously expressed in all tissues with more levels in the brain and skeletal muscle [27–29]. The reduction of the proton motor force across the mitochondrial inner membrane by UCP2 decreased the ROS formation [30,31]. A small reduction in the mitochondrial membrane potential induced by mild uncoupling has a significant effect in attenuating ROS production [32]. Proposed functions of UCP2 include preventing the formation of ROS and atherosclerosis, participation in inflammation, body weight regulation, adaptive thermogenesis and aging [30,33]. Consistent with the protective roles of UCP2 against oxidative stress and inflammation, recent study indicated that pulmonary arterial smooth muscle cells of UCP2-deficient mice showed increased proliferation, MMP and ROS release [34,35]. This seems to be opposite to our findings which showed that genipin decreased VSMC proliferation and migration. One possible explanation is that in addition to inhibit UCP2, genipin increases HO-1 more robustly, which leads to the net effect of genipin to be anti-proliferative and anti-migrative. The physiological outputs of UCP2 inhibition and HO-1 induction by genipin should be carefully dissected in the future study.

**Figure 8:**
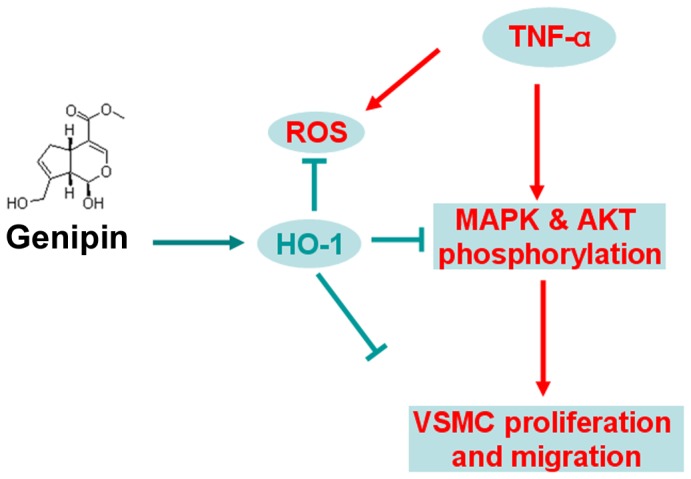
A model illustrating the regulation of VSMC proliferation and migration by genipin through HO-1.

In conclusion, our findings identify for the first time genipin inhibits TNF-α-induced VSMC proliferation and migration. We also demonstrated the roles of HO-1 in anti-inflammatory and anti-oxidative functions of genipin in VSMCs (Figure 8). Genipin may be therefore developed as a novel drug to treat cardiovascular diseases associated with VSMC proliferation and migration such as atherosclerosis. In addition, HO-1 may rank one of the most promising drug targets due to its dynamically modulatory property.

## Materials and Methods

### Reagents

Genipin was purchased from Sigma Aldrich and recombinant rat TNF-α was from Peprotech. For our studies, genipin was dissolved in DMSO and TNF-α were dissolved in 1% BSA. Polyclonal antibody against HO-1 was purchased from Stressgen (Ann Arbor, MI). The antibodies against total ERK1/2, phospho ERK1/2, total Akt, phospho Akt, total p38, phospho p38, total JNK, phospho JNK, cyclin E, PCNA, CDK2, cyclin D1, CDK6 and p27 were obtained from Cell Signaling Technology. The antibodies against CDK4 and p21 were provided by BD Biosciences, and the antibodies against ICAM-1, VCAM-1, OPN, MMP-2, MMP-9, and GAPDH were from Santa Cruz Biotechnology (Santa Cruz, CA). PD98059, Wortmannin, SB203580, SP600125 were purchased from Calbiochem (San Diego, CA).

### Cell culture

VSMCs were isolated from the thoracic aortas of 3 to 4 week-old male SD rats. Cells were used in experiments at passages 4-8. All animal procedures in this investigation conform to the Guide for the Care and Use of Laboratory Animals published by the US National Institutes of Health (NIH publication No. 85-23, revised 1996) and approved by the Laboratory Animal Care Committee at Nanjing University of Chinese Medicine (permit number SYXK 2012-0042). To assess the effects of genipin on cultured VSMCs, we preincubated cells with genipin in serum-free medium and then treated cells with 100 ng/ml TNF-α. For knockdown of HO-1 expression, the rat HO-1 specific siRNA (5’-CCG UGG CAG UGG GAA UUU AUG CCA U-3’) and nonspecific siRNA (5’-CCG ACG GUG AGG UUA UAU CGU GCA U-3’) (serving as a negative control) were designed by siRNA TargetFinder software and synthesized by Invitrogen. VSMCs were transfected with siRNA oligos by X-tremeGENE HP transfection reagent (Roche, USA) according to the manufacturer’s instructions. After 24 h transfection, VSMCs were treated as described previously.

### Proliferation assays

MTT assay was used to evaluate VSMC proliferation rate. VSMCs were seeded in 96-well plates (1×10^4^ cells per well) and pretreated with genipin (25 or 100 μM) for 1 h, and then stimulated with or without TNF-α (100 ng/ml) for 24 h in the presence of genipin. After that, MTT (0.2 mg/ml) was added to each well and incubated for 4 h. The supernatant was removed and the formazan crystals were dissolved in DMSO. Cell proliferation was assessed by measuring the absorbance at 550 nm using a microplate reader. MTT analysis was also confirmed by EdU incorporation assays. 10μM EdU was added into medium for 2 h and then fixed with 4% paraformaldehyde. Cells were counterstained with DAPI (Sigma). Signals were visualized by a fluorescence microscope, and the average ratios between EdU-positive [36] and total DAPI-stained nuclei (blue) were counted for statistic analyses.

### Migration assays

TNF-α dependent chemotaxis of VSMCs was assessed by the scratch wound motility assay. For this assay, VSMCs were seeded in 6-well plates (1.5×10^5^ cells per well) and grew to confluence. Cells were then subjected to a 24 h serum deprivation. To help witness the migrated cells clearly, VSMCs were infected with adenoviruses expressing GFP protein at the beginning of serum deprivation. After that, the cells mounted to a reusable template to create a standard wound (<3 mm). The cells were then incubated with genipin (25 or 100μM) for 1 h followed by stimulation with or without TNF-α (100 ng/ml) for 24 h. Wound closure rates were followed with a reference point in the field of the wound at the bottom of the plate by direct microscopic visualization. The procedure permitted photographing the identical spot each time. The remaining cell-free area was determined via microphotography and performed immediately after 24-h injury.

Cell migration was also assessed with a 24-well modified Boyden chamber containing fibronectin-coated polycarbonate membranes (8 μM pore-size, BD Bioscience, USA). Briefly, the lower wells of the chamber were filled with phenol red-free DMEM supplemented with or without 100 ng/ml TNF-α in the presence or absence of genipin. The filters were coated with 50 mg/ml fibronectin and fixed atop the bottom wells. 1×10^5^ per well VSMCs were allowed to migrate for 6 h and non-migrated cells were removed from the upper side of the membrane with cotton swabs. Cells on the lower side of the membrane were stained with Hoechst 33342, and then counted in five randomly selected squares per well with a fluorescence microscope (Nikon, Japan). Data was presented as numbers of migrated cells per field.

### HO-1 activity analysis

HO-1 activity was evaluated by assaying bilirubin production. In brief, VSMCs were lysed and the cell lysates containing equal amount (80 μg) of total protein were incubated with freshly prepared 2 mM hemin and 4.5 mM NADPH (final reactant concentrations, 41.67 μM hemin, 93.75 μM NADPH, total volume 1920 μl); control samples lacked NADPH. Reactions were then run for 20 min at 37 °C in the dark and terminated by quick-freezing vials on dry ice. The samples were scanned with a spectrophotometer (BioTek Synergy 2, BioTek Instruments, Winooski, VT) at wavelengths of 464–530 nm. Bilirubin concentration was calculated based on the change in optical density from 530 to 464 nm, with an extinction coefficient of 40 mM/cm. HO-1 activity was expressed as micromoles of bilirubin per milligram of protein per h.

### ELISA

Culture medium was collected and centrifuged at 2000 rpm for 10 min at 4°C.The concentration of MMP-2 and MMP-9 in the supernatant was measured by using commercial ELISA kits based on the principle of double-antibody sandwich technique.

### RT-qPCR

Total RNA from cultured VSMCs was extracted using Trizol reagent (Invitrogen, Carlesbad, CA). Two micrograms of total RNA was reverse-transcribed into comple- mentary DNA. 18s ribosomal RNA served as an internal control for total complementary DNA content. mRNA levels were quantified by real-time RT-PCR using SYBR premix Ex Taq (Takara, Japan). Samples were amplified using the Mastercycler ep realplex2 system (Eppendorf, Hamburg, Germany). Primer sequences were: 5’-TTTCACCTTCCCGAGCAT-3’ (F) and 5’-GCCTCTTCTGTCACCCTGT-3’ (R) for HO-1; 5’- AAACGGCTACCACATCCAAG-3’ (F) and 5’-CCTCCAATGGATCCTCGTTA-3’ (R) for 18s rRNA; 5’-AGACAGTGGCAGGGATTCAC-3’ (F) and 5’-TTGAACGACCATCGATGAAA-3’ (R) for IL-8; 5’-CCAAGATGCTATGGGATT-3’ (F) and 5’-AAGATGGCGTCCGCAGTA-3’ (R) for PAI-1.

### Western blotting analysis

We lysed cultured VSMCs in RIPA buffer containing 50 mM Tris–HCl (pH 8.0), 150 mM NaCl, 1% NP-40, 1% sodium deoxycholate, 0.1% sodium dodecylsulfate, 0.1 mM dithiothreitol, 0.05 mM phenylmethyl-sufonylfluoride, 0.002 mg/ml aprotinin, 0.002 mg/ml leupeptin, and 1 mM NaVO3. The protein concentration was quantified with Dc protein assay reagent (Bio-Rad, Hercules, CA). We use 10% SDS-PAGE to loaded and separate proteins. Proteins were transferred onto polyvinylidene difluoride membranes (Millipore, USA). The membranes were incubated with appropriate primary antibodies overnight. HRP-conjugated secondary antibodies (Santa Cruz) were applied to visualize and bound to primary antibodies.

### Measurement of ROS generation

ROS generation in VSMCs was measured by loading cells with 10 μM MitoTracker Red probe (CM-H2XRos) (Invitrogen, USA) for 30 min. The dye solution was freshly prepared in pre-warmed serum-free DMEM medium. After dye loading at 37°C was completed, cells were rinsed twice with phosphate-buffered saline and then stained with DAPI for another 5 min. Finally, cells were washed again for 3 times. The cultures were photographed with a fluorescence microscope (Nikon, Japan, Ti–S 533665).

### Data analysis

Groups of data are presented as mean ± standard error. Data were analyzed using one-way ANOVA followed by Fisher’s LSD post-hoc test. Calculations were performed using SPSS for Windows version 12.5S statistical package (SPSS, Chicago, USA). A value of *P*<0.05 was considered statistically significant.

## Supporting Information

Figure S1 **Validation of the knockdown efficiency of siRNA against HO-1 in Figure 3D.** Cells were treated as Figure 3, HO-1 protein expression level was determined by Western blot.
(TIF)

Figure S2 **Cell viability analysis of adenoviruses expressing GFP.** VSMCs were transfected with/without adenoviruses expressing GFP for 48 h. MTT assay was performed to evaluate the cell viability.
(TIF)

Figure S3 **Determination of HO-1 protein expression levels in the settings of genipin alone or in combinations of various signaling pathway inhibitors.** Cells were pre-incubated with inhibitors for 40 min and then treated with 100 µM genipin for 24 h in the presence of inhibitors. Concentrations: PD98059, 20 μM; Wortmannin, 30 μM; SB203580 and SP600125, 10 μM.
(TIF)
