# Supplementary figures and images for: Genipin Inhibits TNF-α-Induced Vascular Smooth Muscle Cell Proliferation and Migration via Induction of HO-1
Source: PLoS One. 2013 Aug 27;8(8):e74826. doi: 10.1371/journal.pone.0074826 (PMC3754946; doi:10.1371/journal.pone.0074826)

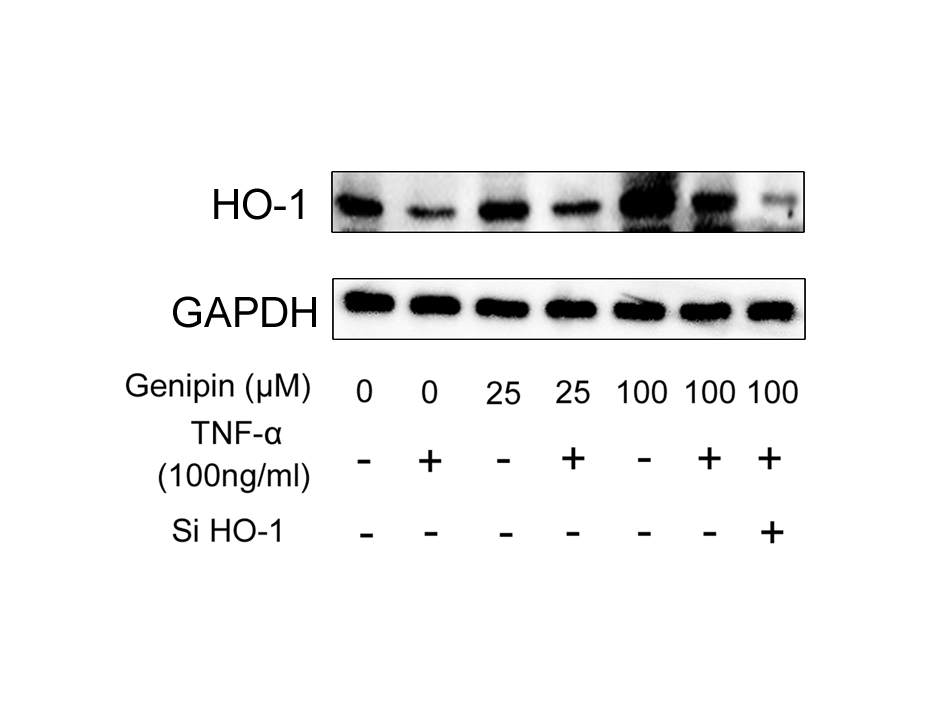

Supplement: Figure S1 — Validation of the knockdown efficiency of siRNA against HO-1 in Figure 3D. Cells were treated as Figure 3, HO-1 protein expression level was determined by Western blot. (TIF) [file pone.0074826.s001.tif]

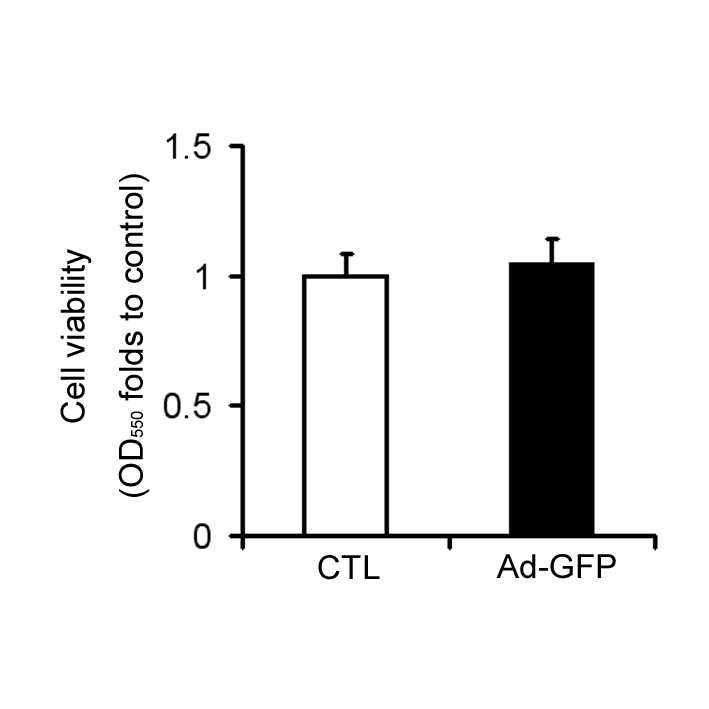

Supplement: Figure S2 — Cell viability analysis of adenoviruses expressing GFP. VSMCs were transfected with/without adenoviruses expressing GFP for 48 h. MTT assay was performed to evaluate the cell viability. (TIF) [file pone.0074826.s002.tif]

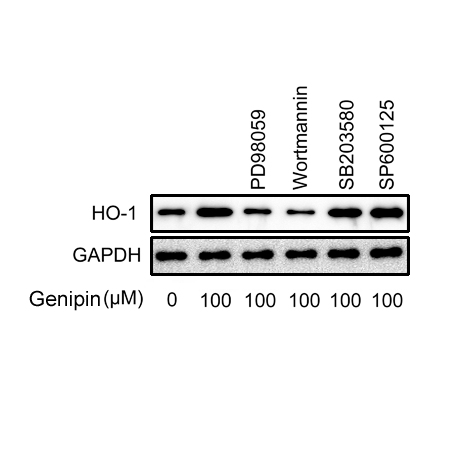

Supplement: Figure S3 — Determination of HO-1 protein expression levels in the settings of genipin alone or in combinations of various signaling pathway inhibitors. Cells were pre-incubated with inhibitors for 40 min and then treated with 100 µM genipin for 24 h in the presence of inhibitors. Concentrations: PD98059, 20 μM; Wortmannin, 30 μM; SB203580 and SP600125, 10 μM. (TIF) [file pone.0074826.s003.tif]
